# Zoonoses and marginalised infectious diseases of poverty: Where do we stand?

**DOI:** 10.1186/1756-3305-4-106

**Published:** 2011-06-14

**Authors:** David Molyneux, Zuhair Hallaj, Gerald T Keusch, Donald P McManus, Helena Ngowi, Sarah Cleaveland, Pilar Ramos-Jimenez, Eduardo Gotuzzo, Kamal Kar, Ana Sanchez, Amadou Garba, Helene Carabin, Amal Bassili, Claire L Chaignat, Francois-Xavier Meslin, Hind M Abushama, Arve L Willingham, Deborah Kioy

**Affiliations:** 1 Centre for Neglected Tropical Diseases, Liverpool School of Tropical Medicine, Pembroke Place, Liverpool, L3 5QA, UK; 2 WHO/EMRO (Eastern Mediterranean Regional Office) Consultant, Communicable Disease Control, c/o Elkoba Street, Apartment 52, Roxy, Cairo, Egypt; 3 National Emerging Infectious Diseases Laboratories and Director, Collaborative Core Special Assistant to the President for Global Health, Boston University, Cross-town Center 391, 801 Massachusetts Avenue, Boston, USA; 4 Molecular Parasitology Laboratory, Queensland Institute of Medical Research, 300 Herston Road, QLD Q 4029, Brisbane, Australia; 5 Department of Veterinary Medicine and Public Health, Sokione University of Agriculture, Mail Box 3021, Morogoro, Tanzania; 6 Institute of Biodiversity, Animal Health and Comparative Medicine, College of Medicine, Veterinary Medicine and Life Sciences, University of Glasgow, Glasgow G12 8QQ, UK; 7 Philippine NGO Council on Population Health and Welfare, No. 304 Diplomat Condominium Bldg, Russel Avenue corner, Roxas Blvd., 1300 Pasay City, Philippines; 8 Instituto de Medicina Tropical "Alexander von Humboldt", Universidad Peruana Cayetano Heredia, Av. Honorio Delgado 430-Urb. Ingenieria-SMP-Lima 3 31 Lima, Peru; 9 R-109, the Residency, City Centre, Salt Lake City, Calcutta 700 064, India; 10 Department of Community Health Sciences, Brock University, 500 Glenridge Avenue, ON L2S 3A1, St. Catharines, Ontario, Canada; 11 Riseal - Niger, 333, avenue des Zarmakoye, BP. 13724, Niamey, Niger; 12 The University of Oklahoma Health Sciences Center, 801 Northeast 13th Street, Room 309AB, Post Office Box 26901, Oklahoma 73104, USA; 13 ZOOM-IN Focal Point, TB Surveillance Officer, Tropical Disease Research, Communicable Disease Control, World Health Organization, Eastern Mediterranean Regional Office, Abdul Razzak Al Sanhouri Street, P.O. Box 7608, Nasr City Cairo 11371, Egypt; 14 Sanitation and Hygiene, Protection of the Human Environment, World Health Organization, 20 Avenue Appia, 1211 Geneva, Switzerland; 15 Zoonoses and Veterinary Public Health, World Health Organization, 20 Avenue Appia 1211 Geneva, Switzerland; 16 Department of Zoology, Faculty of Science, University of Khartoum, P.O. Box 321 11115 Khartoum, Sudan; 17 Special Programme for Research and Training in Tropical Diseases (TDR) World Health Organization, Avenue Appia 20, 1211 Geneva 27, Switzerland

## Abstract

Despite growing awareness of the importance of controlling neglected tropical diseases as a contribution to poverty alleviation and achieving the Millennium Development Goals, there is a need to up-scale programmes to achieve wider public health benefits. This implementation deficit is attributable to several factors but one often overlooked is the specific difficulty in tackling diseases that involve both people and animals - the zoonoses. A Disease Reference Group on Zoonoses and Marginalised Infectious Diseases (DRG6) was convened by the Special Programme for Research and Training in Tropical Diseases (TDR), a programme executed by the World Health Organization and co-sponsored by UNICEF, UNDP, the World Bank and WHO. The key considerations included: (a) the general lack of reliable quantitative data on their public health burden; (b) the need to evaluate livestock production losses and their additional impacts on health and poverty; (c) the relevance of cross-sectoral issues essential to designing and implementing public health interventions for zoonotic diseases; and (d) identifying priority areas for research and interventions to harness resources most effectively. Beyond disease specific research issues, a set of common macro-priorities and interventions were identified which, if implemented through a more integrated approach by countries, would have a significant impact on human health of the most marginalised populations characteristically dependent on livestock.

## Introduction

Infectious diseases disproportionately affect poor and marginalised populations which are subjected to a cycle of ill-health and poverty. With 60% of human infectious diseases caused by zoonotic pathogens [1] effective public health policy must recognise the importance of interactions between humans and animals [2]. The control of neglected tropical diseases (NTDs) for poverty alleviation has become an increasing priority [3,4], but endemic zoonotic diseases are still largely ignored by public health and veterinary services, despite causing a substantial health burden [2,5]. In contrast, for zoonotic diseases with pandemic potential, such as avian or swine influenza and SARS, the international community has responded vigorously with committed resources, reflecting concerns of potential consequences for higher-income countries.

Many endemic zoonoses have a dual impact on human health and livestock production. Human populations dependent on livestock are not only most at direct risk from zoonotic disease but are most vulnerable to the indirect impacts on health of reduced production on livelihoods and food security, which exacerbates the poverty cycle. It is estimated that over 600 million people globally are livestock-dependent, and represent up to 70% of the population in the most marginal areas [6]. These communities are typically isolated from political processes, communication, education and health care, due to geographic, economic and socio-cultural factors, which exacerbate problems of awareness and health-care delivery.

Effective surveillance and control of zoonotic diseases usually requires multisectoral collaboration involving the human health, veterinary, agricultural, educational, wildlife and environment and sanitation sectors. It remains a considerable challenge to coordinate these different interests, and to achieve collaboration in policies, priorities, resourcing and communication at the national and international levels.

Over the last decade significant work on zoonoses has been undertaken; as a result policy has been articulated on prevention and control of individual neglected zoonotic diseases as a generic concept by WHO and partners at three meetings convened since 2005 [2].

**Figure 1:**
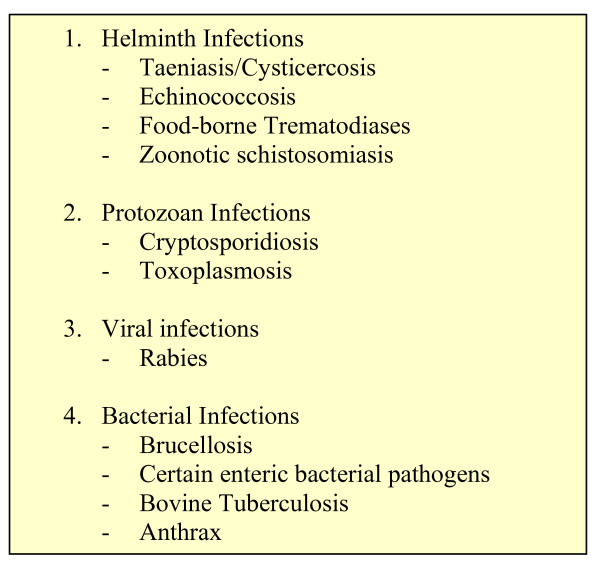
**DRG6 targeted diseases**.

In 2009 WHO UNDP World Bank Special Programme as part of its stewardship function established a Disease Reference Group to address with stakeholders' priority research issues for Zoonotic Diseases and other marginalized infections of poverty (Figure 1). This paper summarises the major findings of DRG6.

### Burden of disease

There are four reasons why zoonotic diseases have been even more neglected than other neglected tropical diseases. Firstly, there is a lack of reliable qualitative and quantitative data on disease burden in endemic countries. This must go beyond the traditional disability-adjusted life year (DALY) assessment to measure and incorporate monetary and health burdens resulting from production losses due to disease in animals [5].

Secondly, clinicians and policy makers often have little knowledge of zoonotic causes of human disease [7] that can be confused with more widely recognized causes of common syndromes including febrile illness, or respiratory or diarrhoeal diseases. Zoonoses presenting as non-specific febrile illness, for example brucellosis, [8] leptospirosis, [9] rickettsiosis and Q-fever, [10] are often misdiagnosed as malaria [11,12]. There is also surprisingly little awareness that long-term sequelae of zoonoses include cancer (food borne trematodiases) or neurological disorders (neurocysticercosis).

Thirdly, the diagnosis of many endemic zoonoses requires capacities that may only be available in referral hospitals and reference laboratories, if at all [13]. For zoonotic schistosomiasis, neurocysticercosis, echinococcosis, opisthorchiasis and clonorchiasis, useful diagnostic imaging techniques are either unavailable or too expensive; similarly, early diagnosis of many bacterial zoonoses depends on sophisticated microbiological or molecular diagnostic methods typically not available to those at greatest risk.

Fourthly, data collection systems for zoonotic diseases are often fragmentary, collected independently by the public health, veterinary or wildlife sectors and recorded and reported separately, if they are recorded at all, resulting in a failure to identify disease outbreaks. They often occur in environments beyond the reach of formal health facilities, education systems and livestock services. Hence, reporting and certification of deaths, a prerequisite for accurate disease burden estimates, often do not exist, contributing to limited awareness and lack of interest and political will to study and control these diseases.

Standardised measures of public health burden, such as the DALY, are only currently available for some of the endemic zoonoses, such as cysticercosis, [14] echinococcosis, [15] human African trypanosomiasis [16] and rabies [17]. Such data have contributed to policy change but information on chronic impacts and nonspecific sequelae of untreated zoonoses remains inadequate. A new metric that incorporates social and economic outcomes is needed to assess the societal impact of zoonotic diseases, and provide the evidence base for objective decision-making and priority-setting.

### Intervention and control of endemic zoonotic diseases

Community-led approaches that empower families and communities to assume responsibility for aspects of disease control can result in feasible and cost-effective strategies to control and, in some cases, eliminate endemic zoonoses. Experience from other neglected disease programmes demonstrates the success of these approaches. For example, the African Program for Onchocerciasis Control (APOC), directly involves communities in decision-making, implementation and monitoring of mass drug administration programmes [18]. Community-led Total Sanitation (CLTS) is another innovative strategy for mobilising communities to completely eliminate open defecation, with sustainable impacts on enteric diseases [19]. Empowering marginalised communities through community-directed interventions offers great promise for tackling endemic zoonoses, and should be encouraged and supported by local and international technical and financial resources.

Despite these successes, an intervention vacuum still exists for many zoonoses - even when the outcome and cost-effectiveness of interventions are known - because of entrenched perceptions of health impacts and priorities. Local neglect is sometimes exacerbated by international disregard, with international priorities focusing on diseases that pose an emerging global threat, such as influenza A H5N1, but are of limited importance to impoverished communities in comparison to endemic zoonoses. Despite the promise of the Alma Ata Declaration [20] to attain 'health for all' by the year 2000, marginalised communities still suffer from poor access to health technologies and services, which continues to undermine all disease control efforts.

### "One Health"

The 'One Health' philosophy, to forge inclusive collaborations between human and animal health professionals, and related environment and agricultural disciplines, currently dominates much of the discussion of zoonotic diseases. While the concept in theory has been widely embraced progress in practice to ensure genuine integration lags behind, not only across academic disciplines, but also with respect to integration of research with policy. Too often research questions are formulated without input from policy-makers, when effective 'buy-in' could be achieved by integration and iterative engagement throughout the research development cycle [21-23].

A clear advantage of One Health is that interventions in animal populations can result in public health and societal benefits more cost-effectively than just interventions in humans. For example, although human rabies can be prevented through timely post-exposure prophylaxis, the high cost of human vaccination places a significant burden on health budgets, in contrast to mass vaccination of domestic dog reservoirs. Similarly, a comprehensive control strategy in China based on interventions to reduce the rate of transmission of *Schistosoma japonicum *infection from bovines and humans to snails has been highly effective, [24] and has now been adopted by the Chinese government as the national strategy for the control of schistosomiasis. In Uganda, sleeping sickness caused by *Trypanosoma rhodesiense *is being controlled by the mass chemotherapy treatment of the cattle reservoir and insecticidal treatment to control tsetse populations which also reduces tick populations [5,16].

Integrated, trans-disciplinary approaches envisioned under One Health are more likely to be adopted when they provide added value. Many opportunities exist for adding value through shared resources and expertise, for example, in zoonotic disease surveillance. Investments to enhance laboratory capacity to diagnose avian influenza provided a useful opportunity to enhance the surveillance of other zoonotic diseases but there are few examples of this for endemic zoonoses. The widespread perception that testing human and animal samples must be conducted in separate laboratory facilities, for which there is little rationale, increases the cost for diagnostic facilities, and is a major barrier to integration of disease surveillance efforts between different Ministries.

Whereas One Health aims to expand our thinking beyond the confines of disciplinary silos, the way forward will not necessarily be straightforward. Traditional roles and responsibilities may need to be relinquished while financial control is shared or ceded entirely to another sector. But, potential health gains for the most impoverished surely make these changes worth pressing for.

### Macro research priorities and recommendations to policy makers

**Figure 2:**
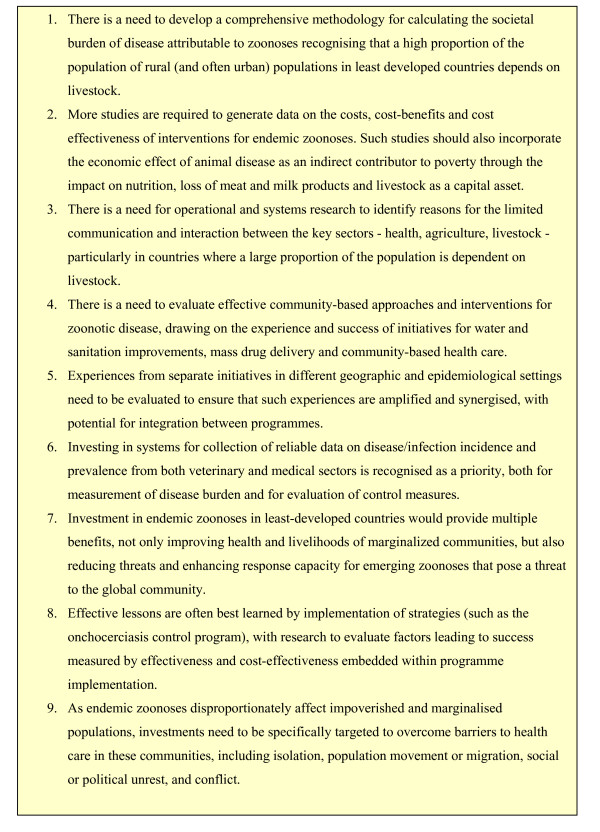
**Macro research priorities identified by DRG6**.

DRG6 identified a set of macro-priorities for facilitating interactions between applied researchers to promote necessary intervention research on zoonotic diseases of marginalised populations (Figure 2).

Investments in disease burden assessments for zoonotic diseases are essential to provide an advocacy base to highlight their importance. A prerequisite is country prioritisation and commitment from different sectors, including finance, national research institutions, and political commitment to stable policy supplemented by long term international support. This will allow development of national guidelines that establish and sustain veterinary public health units, and clarify their role in tandem with the human health system. These will improve public health care for, and actually beyond, the neglected zoonotic diseases.

## List of Abbreviations

**A H5N1**: Highly pathogenic avian influenza; **APOC**: African Program for Onchocerciasis Control; **CLTS**: Community-led Total Sanitation; **DALY**: Disability-adjusted life year; **DRG6**: Disease Reference Group on Zoonoses and Marginalised Infectious Diseases; **GSK**: GlaxoSmithKline; **TDR**: The Special Programme for Research and Training in Tropical Diseases; **UNDP**: United Nations Development Programme; **UNICEF**: The United Nations Children's Fund; **WHO**: World Health Organization

## Competing interests

DHM is a Senior Professorial Fellow in the Center for Neglected Tropical Diseases, in Liverpool Tropical of Medicine, which receives funding from the UK Department for International Development and GlaxoSmithKline (GSK) in support of the Global Programme and Global Alliance for the Elimination of Lymphatic Filariasis. He also has a consulting agreement with Pfizer and is Chair of WHO/TDR Disease Reference Group on Zoonoses and other marginalized infectious diseases of poverty.

All other authors declare that they have no Conflict of Interest

## Authors' contributions

GK drafted the first version of the manuscript based on the work of the whole Disease Reference Group which was subsequently redrafted and edited with additional material from DHM, DPM, SC and DK and further reviewed by ZH. All members of the Group reviewed the final manuscript and their signatures are below
